# Inhibition of HDAC6 Activity Alleviates Myocardial Ischemia/Reperfusion Injury in Diabetic Rats: Potential Role of Peroxiredoxin 1 Acetylation and Redox Regulation

**DOI:** 10.1155/2018/9494052

**Published:** 2018-06-25

**Authors:** Yan Leng, Yang Wu, Shaoqing Lei, Bin Zhou, Zhen Qiu, Kai Wang, Zhongyuan Xia

**Affiliations:** Department of Anesthesiology, Renmin Hospital of Wuhan University, Wuhan, 430060 Hubei Province, China

## Abstract

Patients with diabetes are more vulnerable to myocardial ischemia/reperfusion (MI/R) injury, which is associated with excessive reactive oxygen species (ROS) generation and decreased antioxidant defense. Histone deacetylase 6 (HDAC6), a regulator of the antioxidant protein peroxiredoxin 1 (Prdx1), is associated with several pathological conditions in the cardiovascular system. This study investigated whether tubastatin A (TubA), a highly selective HDAC6 inhibitor, could confer a protective effect by modulating Prdx1 acetylation in a rat model of MI/R and an *in vitro* model of hypoxia/reoxygenation (H/R). Here, we found that diabetic hearts with excessive HDAC6 activity and decreased acetylated-Prdx1 levels were more vulnerable to MI/R injury. TubA treatment robustly improved cardiac function, reduced cardiac infarction, attenuated ROS generation, and increased acetylated-Prdx1 levels in diabetic MI/R rats. These results were further confirmed by an *in vitro* study using H9c2 cells. Furthermore, a study using Prdx1 acetyl-silencing mutants (K197R) showed that TubA only slightly attenuated H/R-induced cell death and ROS generation in K197R-transfected H9c2 cells exposed to high glucose (HG), but these differences were not statistically significant. Taken together, these findings suggest that HDAC6 inhibition reduces ROS generation and confers a protective effect against MI/R or H/R injury by modulating Prdx1 acetylation at K197.

## 1. Introduction

Diabetes mellitus (DM) is one of the most important risk factors for developing coronary heart disease [1]. Moreover, the mortality rate is significantly higher among patients with DM than among those without DM who present with acute myocardial infarction (AMI) [2]. Timely revascularization is key to improving AMI outcomes. However, reperfusion induces further damage, which is largely attributed to reactive oxygen species (ROS) that accumulate during the early reperfusion phase [3–5]. Intracellular hyperglycemia-induced ROS production and decreased antioxidant defenses may render diabetic hearts more vulnerable to MI/R injury [6]. Researchers have found that ROS scavengers can reduce the MI/R-induced infarct size, which suggests that the response to MI/R can be manipulated by eliminating ROS to alleviate injury [7–9]. However, the cellular mechanisms controlling ROS production and scavenging are not fully understood.

ROS are by-products of mitochondrial respiration, and the primary ROS produced by the mitochondria is superoxide (O_2_^−^). The majority of O_2_^−^ undergoes dismutation to become hydrogen peroxide (H_2_O_2_) [10]. H_2_O_2_ is reduced by antioxidant systems, some of which lead to the formation of hydroxyl radicals (OH^•^) via the Fenton reaction [11]. Excessive OH^•^ oxidizes lipids, proteins, and nucleic acids, thus impairing cellular function and cardiovascular pathology [12]. Therefore, reducing H_2_O_2_ generation or accelerating its decomposition may contribute to ameliorating MI/R injury.

Histone deacetylases (HDACs) play an essential role in both epigenetics and signaling modification by catalyzing the removal of acetyl groups from lysine residues of histone and nonhistone proteins [13]. Among eighteen identified HDACs in mammals, HDAC6 is unique for its predominantly cytoplasmic localization and two catalytic sites [14]. HDAC6 regulates various cellular processes, including autophagy, microtubule-dependent transport, and cell migration, by deacetylating nonhistone proteins, such as *α*-tubulin, cortactin, and HSP90 [15–17]. Accumulating evidence suggests that HDAC6 is involved in redox regulation and cellular stress responses. For example, HDAC6 inhibitor attenuates renal tubular damage and reduces apoptotic cell death by suppressing oxidative stress in acute kidney injury (AKI) mice [18]. Additionally, oxidized low-density lipoprotein (LDL) induces oxidative injury in endothelial cells, which can be prevented by HDAC6 inhibition [19]. HDAC6 was recently found to play a critical role in the pathological processes of cardiovascular disease. HDAC6 was also previously shown to be consistently increased in stressed myocardium [20]. Moreover, inhibition of HDAC6 preserves systolic function in angiotensin-II- or transverse aortic constriction-induced pressure-overloaded hearts [21, 22]. These studies collectively suggest that HDAC6 is a promising therapeutic target for reducing oxidative stress in myocardium. However, the protective mechanisms of HDAC6 inhibition are not fully understood.

A recent research study showed that the acetylated form of peroxiredoxin 1 (Prdx1) was accumulated in HDAC6 knockout cells; thus, Prdx1 was thought to be a specific target of HDAC6 [23]. Prdx1 is an antioxidant protein, as its specialized cysteine residues can break down intracellular peroxide [24]. Previous studies have shown that Prdx1 acetylation elevates its peroxide-reducing activity [23, 25]. Moreover, Prdx1 is ubiquitous and highly expressed in myocardium [26]. Therefore, increasing Prdx1 acetylation is critical for ROS elimination.

In this study, we aimed to identify the possible beneficial effects of HDAC6 inhibition on streptozotocin- (STZ-) induced diabetic rats following MI/R and investigated whether HDAC6 inhibition can increase ROS elimination by modulating Prdx1 acetylation.

## 2. Methods

### 2.1. Experimental Animals and Diabetes Induction

Sixty male Sprague-Dawley (SD) rats (220 ± 10 g) were supplied by Huafukang Bioscience (Beijing, China). Rats were housed in an environment with a maintained temperature and relative humidity and a fixed light/dark schedule (12 h light/12 h dark). All rats were given free access to standard chow and water. After 5 days of acclimatization, the rats were fasted for 12 h for diabetes induction. Type 1 diabetic rats were induced by a single intraperitoneal (i.p.) injection of 65 mg/kg STZ (Sigma-Aldrich) as previously described [27, 28]. Rats in the nondiabetic group were injected with an equal volume of sodium citrate buffer. Rats exhibiting hyperglycemia (blood glucose level higher than 16.7 mmol/l) were considered to have diabetes. All animal procedures were in accordance with the Principles of Laboratory Animal Care of Wuhan University and were approved by the Committee for the Use of Live Animals in Teaching and Research.

### 2.2. MI/R Injury Model

We used a well-established model of MI/R injury. In brief, rats were anesthetized by i.p. injection of pentobarbital sodium (50 mg/kg). We continuously monitored the electrocardiogram (ECG) and heart rate (HR) by an electrophysiolograph (BioPAC, MH150, USA). The heart was exposed by a thoracotomy and pericardiotomy, and MI/R was achieved by occluding the left anterior descending (LAD) coronary artery for 45 min followed by reperfusion for 3 h. The sham-operated group underwent the same surgical procedure without LAD coronary artery occlusion. After reperfusion, the animals were sacrificed to collect blood and tissue samples for subsequent experiments.

### 2.3. Experimental Protocols

For the *in vivo* study, 8 weeks after STZ administration, diabetic and normal rats were randomly assigned to one of the following four groups: (1) normal + sham group (NS); (2) normal + I/R group (NIR); (3) DM + sham group (DS); and (4) DM + I/R group (DIR). Furthermore, to evaluate the cardioprotective effects of tubastatin A (TubA) and the role of HDAC6 and Prdx1 in this process, a second set of experiments was performed on the following groups: (1) DM + sham group (DS); (2) DM + I/R group (DIR); and (3) DM + TubA group (DIR + TubA). TubA (10 mg/kg) or vehicle (DMSO, 1‰) was intraperitoneally administered for 7 days before MI/R injury. For the *in vitro* study, H9c2 cardiomyocytes were randomly assigned to the following groups: (1) high glucose control (HG, 33 mM glucose), (2) cells exposed to high glucose followed by H/R (HGHR), and (3) cells exposed to HGHR with TubA pretreatment (HGHR + TubA). We next tested whether the K197 residues of Prdx1 were at the acetylation site of HDAC6. A second set of experiments was performed in Prdx1-WT-HA- and Prdx1-K197R-HA-transfected H9c2 cells, which were divided into the following groups: (1) HG control (HG), (2) cells exposed to HGHR (HGHR), and (3) cells exposed to HGHR with TubA pretreatment (HGHR + TubA).

### 2.4. Left Ventricular Function

Myocardial function was intermittently monitored by invasive hemodynamic measurements. Data were collected at baseline and at 0, 60, 120, and 180 min of reperfusion. The method has been previously described [28, 29]. In brief, a heparin-saline-filled catheter was inserted into the left ventricle via an incision in the right common carotid artery. The other end of the catheter was connected to a pressure transducer (Yixinda, Shenzhen, China). Left ventricular systolic pressure (LVSP) and the maximal rates of the increase and decrease in LVSP (±dp/dtmax) were monitored by an electrophysiolograph (BioPAC). Data processing was performed using AcqKnowledge 4.0 software.

### 2.5. Determination of Myocardial Infarct Size

After MI/R, rats were sacrificed to assess the sizes of the infarct area (IA) and area at risk (AAR). The method has been previously described [29]. In brief, the LAD ligature was retied, and 2 ml of 2% Evans Blue dye (Sigma, USA) was injected into the femoral vein immediately after 3 hours of reperfusion (*n* = 6 rats/group). Then, rats were sacrificed, and their hearts were excised. The IA was measured by 2,3,5-triphenyltetrazolium chloride staining (Sigma-Aldrich). The infarct size was determined by using an image analysis system (Image-Pro Plus 3.0; Media Cybernetics, MA). Two researchers independently scored the heart slides to ensure reliability of the results. The percentage of IA versus AAR (IA/AAR × 100%) was calculated.

### 2.6. Measurement of Lactate Dehydrogenase and Creatine Kinase-MB Activity

For the *in vivo* study, arterial blood of each rat was collected at the end of reperfusion and fuged to collect the serum (2000 rpm, 10 min). For the *in vitro* study, cell culture supernatant was collected to measure the release of lactate dehydrogenase (LDH), after the HG and H/R insult. Levels of cardiovascular biomarkers, including creatine kinase-MB (CK-MB) and LDH, were measured using a commercially available kit (Beyotime Biotechnology, China) according to the manufacturer's instructions.

### 2.7. Immunoprecipitation and Western Blot Analysis

Protein levels in left ventricular tissue or cultured cells were measured as described in [29]. Primary antibodies against HDAC6, Prdx1, Acetyl-K, HA, and GAPDH (1 : 1000 dilution, Cell Signaling Technology) were used. GADPH served as a loading control to ensure equal loading. Optical densities of signals were quantified by a fluorescence imaging scanner (Odyssey, Germany). Western blotting assays were repeated six times for each group with myocardial tissues or cell cultures. After MI/R or H/R, cardiac tissues or cells were washed with cold PBS, and the IP lysates were harvested. A 200 *μ*g sample of cell or tissue lysate was subjected to immunoprecipitation with 1 *μ*g of anti-Prdx1 or anti-HA antibody in the presence of 20 *μ*l of protein A/G plus-agarose by rotating overnight at 4°C. The protein G sepharose complex was purified and eluted with IP buffer. Immunoprecipitants or the input sample (total lysate) was resolved by SDS–PAGE and subjected to Western blot analysis.

### 2.8. Determination of Apoptosis

For the *in vivo* study, MI/R-induced apoptosis was evaluated by using an in situ terminal deoxynucleotidyl nick-end labeling (TUNEL) assay. The left ventricle of each group was removed and incubated in 4% paraformaldehyde overnight at room temperature after MI/R. The apoptosis rate was determined according to the manufacturer's protocol (Roche, Indianapolis, USA). Ten fields for each sample were randomly chosen to determine the apoptosis rate. For the *in vitro* study, after H/R injury, H9c2 cells were collected and resuspended with binding buffer. Cells were incubated with fluorescein isothiocyanate-conjugated annexin V (FITC-annexin V) and propidium iodide (PI) in the dark for 10 minutes. The fluorescence signal of each group was measured by using a FACSCalibur instrument (BD Biosciences, USA). The data obtained from the H9c2 cell population were analyzed using CellQuest Pro software (BD Biosciences, USA).

### 2.9. Cell Culture and Transfection

H9c2 cardiomyocytes were cultured in low-glucose DMEM with 10% FBS at 37°C in a humidified atmosphere of 10% CO_2._ When cells in the six-well plates reached 60–70% confluence, they were incubated in serum-free DMEM containing 0.1% BSA overnight. Cells were exposed to HG medium for 24 h. Cells in the TubA-treated group were incubated with medium containing TubA (5 *μ*M) for 24 h before H/R, and the cardiomyocytes were then subjected to 4 h of hypoxia (94% N_2_ and 5% CO_2_) and 2 h of reoxygenation. Two HA-tagged Prdx1 constructs, Prdx1-WT-HA and Prdx1-K197R-HA, and another nontagged vector were used. Prdx1-K197R-HA and Prdx1-WT-HA mutants were generated by using a QuikChange II site-directed mutagenesis kit (Stratagene) according to the manufacturer's instructions. For cell transfection, plasmids were mixed with Lipofectamine 2000 (Invitrogen, USA) in Opti-MEM (Gibco, USA) and transfected into H9c2 cells according to the manufacturer's instructions. After H/R, the cells and the culture medium were collected and stored separately for analysis. Each experiment was performed at least six times.

### 2.10. HDAC6 Activity

HDAC6 activity was measured using a HDAC6 fluorometric activity assay kit (Biovision, CA, USA). In brief, lysates of myocardial tissue or H9c2 cells were suspended in assay buffer and incubated with a synthetic acetylated-peptide substrate of HDAC6 for 1 h at 37°C. The lysine developer produced a fluorophore, which was quantified by using a fluorescence plate reader.

### 2.11. Mitochondrial Permeability Transition Pores (mPTPs) in Cardiomyocytes

To examine mPTP opening, an mPTP fluorescence assay (Genmed Scientifics Inc., MA, USA) was performed. This method has been previously described [29]. In brief, H9c2 cells were loaded with 8 mM cobalt chloride and 0.25 mM calcein-acetoxymethylester (calcein-AM) at 37°C for 20 min. The fluorescence signal was observed by using a fluorescence microscope (Olympus, Bx 50-FLA) at 488 nm excitation and 525 nm emission. The results are presented as relative fluorescence intensity. The average fluorescence intensity was analyzed by Image-Pro advanced software.

### 2.12. DCFH-DA Assay

Intracellular ROS levels were measured by using commercial DCFH-DA molecular probes (Sigma, USA) as previously described [29]. In brief, cardiomyocytes in six-well plates were loaded with 2 ml of 10 *μ*M DCFH-DA probes for 30 min at 37°C in the dark. DCFH-DA converts into highly fluorescent DCFH upon oxidation and exhibits green fluorescence in the cytosol. Fluorescence images were captured by a fluorescence microscope (Olympus IX51). The mean fluorescence intensity (MFI) was calculated by Image-Pro advanced software. The results represent six independent experiments.

### 2.13. Measurement of O_2_^−^, H_2_O_2,_ Malondialdehyde (MDA), and Lipid Peroxidation Levels

To examine O_2_^−^ production in cardiac tissues or H9c2 cells, the lucigenin chemiluminescence method was utilized. Briefly, after H/R, the supernatant samples were collected and loaded with 5 *μ*M dark-adapted lucigenin; subsequently, light emission was detected by using a luminometer (GloMax, Promega) for 30 min at room temperature. Light emission was recorded every 5 min. The results are expressed as the mean light units (MLU)/min/100 *μ*g protein. H_2_O_2_ content was determined by using a hydrogen peroxide assay kit (Beyotime Institute of Biotechnology, Jiangsu, China) according to manufacturer's instructions. Malondialdehyde (MDA) and lipid peroxidation concentrations were analyzed spectrophotometrically according to the instructions of the assay kits (Nanjing Jiancheng Bioengineering Institute, Nanjing, China).

### 2.14. Cell Viability Assay

Cell viability was determined by cell counting kit-8 (Dojindo, Kumamoto, Japan) in 96-well plates according to manufacturers' instructions. In brief, the CCK-8 solution was added to each well after the treatments and incubated for 3 h. The absorbance of each well was measured at 450 nm by using a microplate reader to calculate the percentage of viable cells.

### 2.15. Statistical Analysis

All data are expressed as the mean ± SEM. Comparisons between multiple groups were made by one-way ANOVA followed by the Tukey test. Statistical analysis was performed using GraphPad Prism 6.0 for Windows (GraphPad Software, USA). *p* < 0.05 was considered statistically significant.

## 3. Results

### 3.1. General Characteristics of the Experimental Animals before MI/R Injury

As shown in Table 1, 8 weeks after STZ injection, type 1 diabetic rats exhibited symptom characteristic of diabetes, including hyperglycemia, polydipsia, polyphagia, weight loss, and increased heart/body weight ratio compared with age-matched nondiabetic rats.

### 3.2. STZ-Induced Diabetic Rats Exhibited Aggravated MI/R Injury and ROS Generation

**Figure 1:**
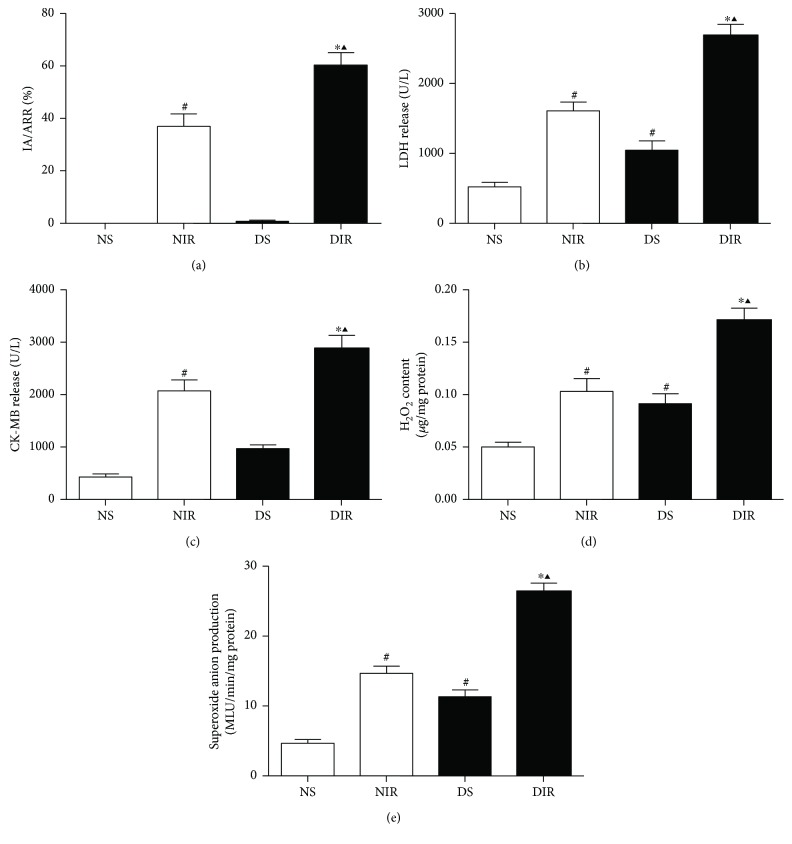
Effects of MI/R injury on nondiabetes and diabetic rats. Nondiabetes and diabetic rats were subjected to 45 min of ischemia followed by 3 h of reperfusion. N: nondiabetic rats; D: STZ-induced diabetic rats; S: sham operation; IR: ischemia/reperfusion; TubA: tubastatin A. (a) Infarct area versus area at risk (IA/AAR × 100%). (b) Serum levels of LDH. (c) Serum levels of CK-MB. (d) Cardiac tissue H_2_O_2_ concentration. (e) Cardiac tissue O_2_^−^ production. All the results are presented as mean ± SEM, *n* = 6/group. ^∗^*p* < 0.05 versus NIR group, ^#^*p* < 0.05 versus NS group, and ^▲^*p* < 0.05 versus DS group.

To investigate the effects of diabetes on MI/R injury, we measured the infarct size, biochemical markers, and ROS generation in the experimental groups. Compared with the NS group, serum LDH (Figure 1(b)) and CK-MB (Figure 1(c)) levels as well as cardiac H_2_O_2_ (Figure 1(d)) and O_2_^−^ (Figure 1(e)) levels were significantly increased in the DS group at baseline. Diabetic rats subjected to MI/R showed larger infarct sizes (Figure 1(a)), higher serum LDH (Figure 1(b)) and CK-MB (Figure 1(c)) levels, and higher cardiac H_2_O_2_ concentration (Figure 1(d)) and O_2_^−^ production (Figure 1(e)) than the nondiabetic rats. These results suggest that diabetic hearts are more vulnerable to MI/R injury.

### 3.3. Diabetes Elevated HDAC6 Activity and Decreased Prdx1 Acetylation

**Figure 2:**
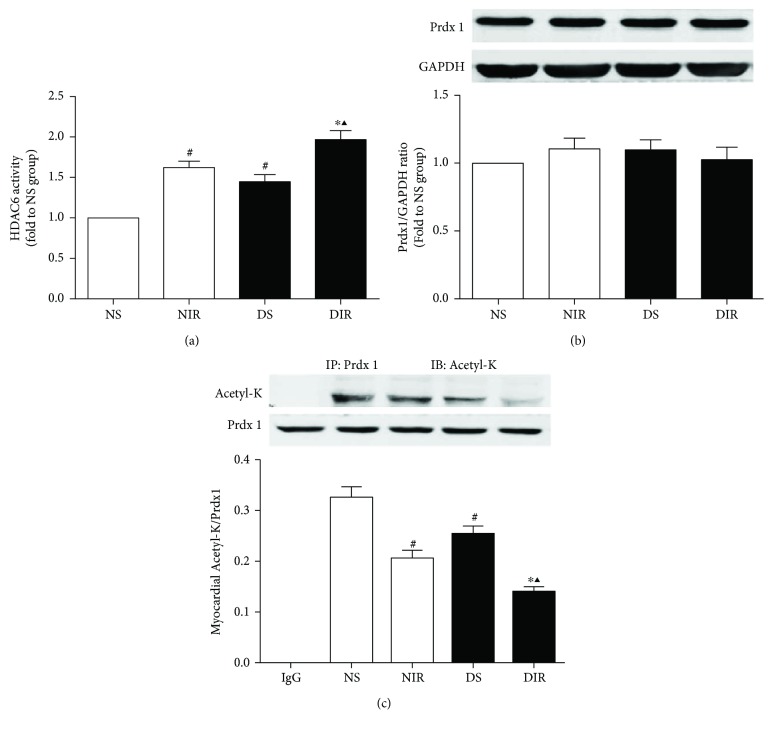
Effects of MI/R injury on HDAC6 activity, total Prdx1, and Ac-Prdx1 in nondiabetes and diabetic rats. Rats were subjected to 45 min of ischemia followed by 3 h of reperfusion. N: nondiabetic rats; D: STZ-induced diabetic rats; S: sham operation; IR: ischemia/reperfusion; TubA: tubastatin A. (a) HDAC6 activity was measured by fluorometric assay kit. (b) Total Prdx1 level of myocardium was analyzed by Western blot. GAPDH served as the loading control. (c) Acetylation level of Prdx1 was analyzed by immunoprecipitation. All the results are presented as mean ± SEM, *n* = 6/group. ^∗^*p* < 0.05 versus NIR group, ^#^*p* < 0.05 versus NS group, and ^▲^*p* < 0.05 versus DS group.

Next, we evaluated HDAC6 activity, total Prdx1 levels, and acetylated-Prdx1 levels in diabetic and nondiabetic rats to explore the underlying mechanism of aggravated MI/R injury in diabetes. Immunoprecipitation experiments were performed to measure the level of Prdx1 acetylation, and the result is presented as acetyl-K/Prdx1 ratios. Compared with the nondiabetic hearts, HDAC6 activity (Figure 2(a)) was increased while Prdx1 acetylation (Figure 2(c)) was decreased in diabetic rats. Moreover, MI/R injury significantly increased HDAC6 activity (Figure 2(a)) but reduced acetyl-K/Prdx1 ratios (Figure 2(c)) in both the diabetic and nondiabetic groups. In addition, there were no significant changes in total Prdx1 levels among NS, NIR, DS, and DIR groups (Figure 2(b)). These results provided evidence that increased HDAC6 activity and decreased levels of acetylated Prdx1 may be responsible for aggravated MI/R injury in diabetes.

### 3.4. Suppression of HDAC6 Activity by TubA Attenuated MI/R-Induced Cell Injury and Cardiac Dysfunction in Diabetic Rats

**Figure 3:**
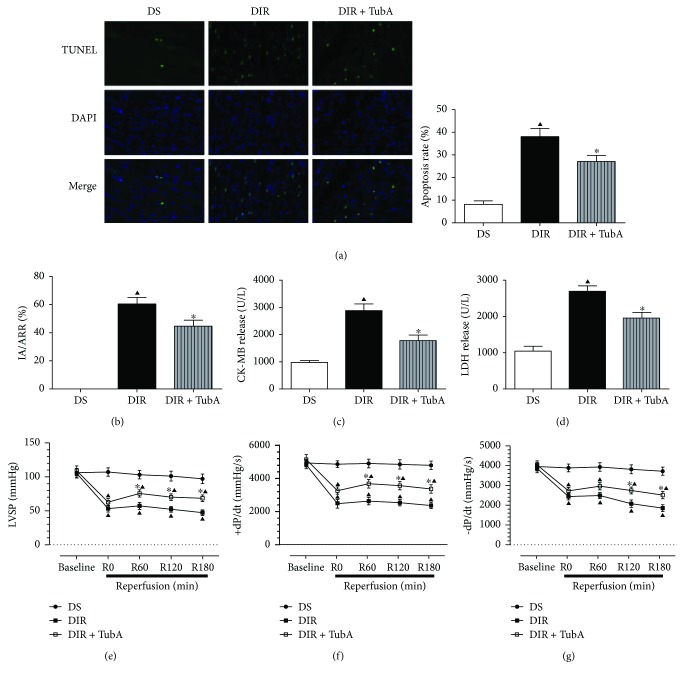
Effects of TubA on apoptosis rate, infract size, serum biomarks, and LV hemodynamic parameters in diabetic rats. Diabetic rats were subjected to 45 min of ischemia followed by 3 h of reperfusion. Tubastatin A (10 mg/kg) was administrated daily by i.p. injection for 1 week in diabetic rats. D: STZ-induced diabetic rats; S: sham operation; IR: ischemia/reperfusion; TubA: tubastatin A. (a) Representative TUNEL staining image and apoptosis rate of each group. (b) Infarct area versus area at risk (IA/AAR × 100%). (c) Serum CK-MB levels. (d) Serum LDH levels. LV hemodynamic parameters including LVSP (e), +dp/dt max (f), and −dp/dt max (g) were measured in each group. All values are presented as the mean ± SEM, *n* = 6/group. ^∗^*p* < 0.05 versus DIR group, ^▲^*p* < 0.05 versus DS group.

We next investigated whether inhibition of HDAC6 activity with TubA could alleviate MI/R injury in diabetic rats. As shown in Figure 3(b), the untreated diabetic rats subjected to MI/R had larger infarct sizes than rats in the TubA treatment group (Figure 3(b)). We also measured the apoptosis rate and biochemical markers of MI/R injury in the experimental rats. MI/R significantly increased the cell apoptosis rate (Figure 3(a)), plasma LDH levels (Figure 3(d)), and CK-MB levels (Figure 3(c)). However, the rats treated with TubA demonstrated significantly decreased cell apoptosis rates and plasma LDH and CK-MB levels compared with DIR rats. Hemodynamic parameters were monitored to evaluate left ventricular function (Figures 3(e)-3(g)). During the MI/R period, all hemodynamic parameters in the experimental rats were significantly decreased compared with baseline. However, TubA treatment recovered the LVSP, +dp/dt at the R60 time point (reperfusion for 60 min), and −dp/dt at the R120 time point (reperfusion for 120 min).

### 3.5. TubA Reduced ROS Generation and Lipid Peroxidation in MI/R Diabetic Hearts

**Figure 4:**
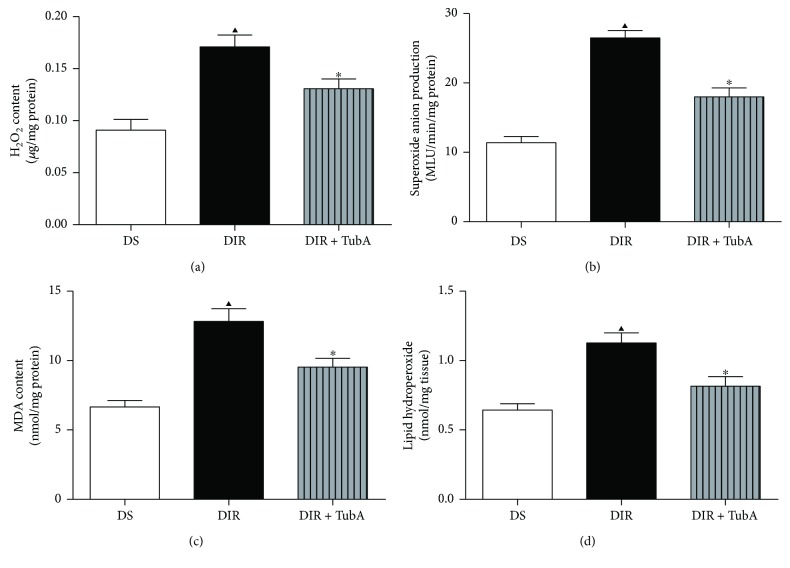
Effect of TubA on oxidative stress in MI/R diabetic rats. After diabetic rats were subjected to 45 min of ischemia followed by 3 h of reperfusion, H_2_O_2_, MDA, lipid hydroperoxide level, and O_2_^−^ production were measured to assess the oxidative stress. D: STZ-induced diabetic rats; S: sham operation; IR: ischemia/reperfusion; TubA: tubastatin A. (a) Cardiac tissue H_2_O_2_ concentration. (b) Cardiac tissue superoxide anion production. (c) Cardiac tissue MDA content. (d) Cardiac tissue lipid hydroperoxide. All values are presented as the mean ± SEM, *n* = 6/group. ^∗^*p* < 0.05 versus DIR group, ^▲^*p* < 0.05 versus DS group.

To identify the possible mechanisms underlying the cardioprotective effects of HDAC6 inhibition on diabetic rats, we evaluated cardiac ROS levels by measuring the H_2_O_2_ concentration (Figure 4(a)) and O_2_^−^ production (Figure 4(b)). We further measured MDA (Figure 4(c)) and lipid hydroperoxide (Figure 4(d)) levels as indicators of oxidative stress. As shown in Figure 4, MI/R-induced ROS generation and oxidative stress, as demonstrated by significantly increased levels of cardiac H_2_O_2_, MDA, lipid hydroperoxide, and O_2_^−^ production. TubA treatment attenuated the MI/R-induced H_2_O_2_ and O_2_^−^ production. Moreover, TubA treatment alleviated ROS-induced oxidative stress, as demonstrated by decreased MDA and lipid hydroperoxide levels. These results collectively suggest that HDAC6 inhibition reduces MI/R-induced ROS generation and oxidative stress in diabetic rats.

### 3.6. Effect of TubA on HDAC6 Activity and Prdx1 Acetylation in MI/R Diabetic Hearts

**Figure 5:**
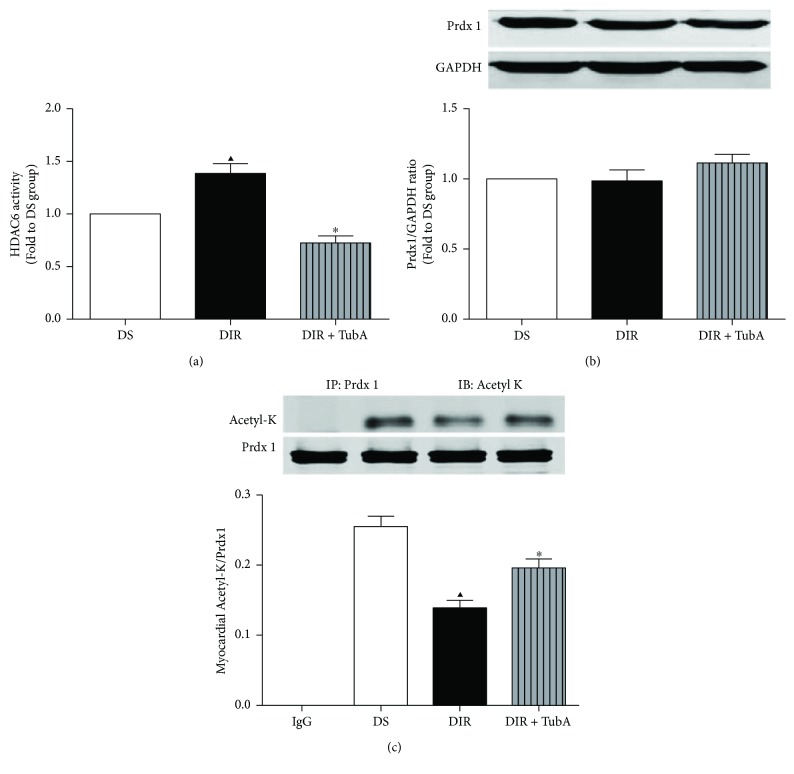
Effect of TubA on HDAC6 activity, total Prdx1, and Ac-Prdx1 in diabetic rats. D: STZ-induced diabetic rats; S: sham operation; IR: ischemia/reperfusion; TubA: tubastatin A. (a) HDAC6 activity was measured by fluorometric assay kit. (b) Total Prdx1 level of myocardium was analyzed by Western blot. GAPDH served as the loading control. (c) Acetylation level of Prdx1 was analyzed by immunoprecipitation. All values are presented as the mean ± SEM, *n* = 6/group. ^∗^*p* < 0.05 versus DIR group, ^▲^*p* < 0.05 versus DS group.

We further investigated whether HDAC6 activity was involved in the regulation of Prdx1 acetylation in diabetic rats. TubA treatment significantly decreased HDAC6 activity, suggesting that TubA is a potent HDAC6 inhibitor (Figure 5(a)). MI/R increased cardiac HDAC6 activity but decreased the level of acetylated Prdx1 (Figure 5(c)) in diabetic rats compared with the sham group. However, TubA treatment significantly increased the acetyl-K/Prdx1 ratio, suggesting that Prdx1 acetylation is regulated by HDAC6 activity. There was no significant difference in the expression of total Prdx1 among the sham, MI/R, and TubA-treated groups (Figure 5(b)).

### 3.7. HDAC6 Inhibition Conferred Protective Effects against H/R Injury in Cultured H9c2 Cells Exposed to HG

**Figure 6:**
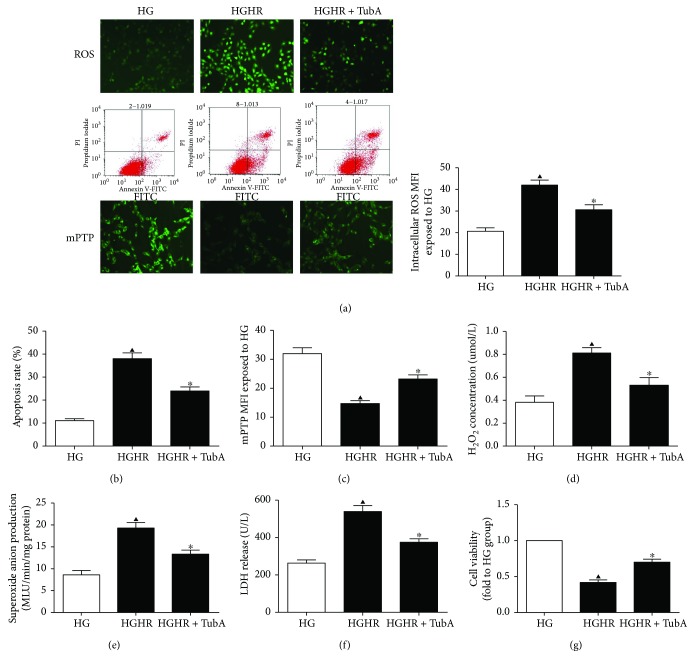
Effects of TubA on ROS, mPTP, apoptosis rate, and cellular injury in H9c2 cells exposed to HG. HG: high glucose (30 mM); HR: hypoxia/reoxygenation; TubA: tubastatin A. H9c2 cardiomyocytes were subjected to 4 h of hypoxia followed by 2 h of reoxygenation with or without TubA under LG or HG stimulation. (a) Representative images of ROS staining and the mean fluorescence intensity of DCFDA in each group. (b) Representative images of flow cytometry and cardiomyocyte apoptosis rates in each group. (c) Fluorescent images of the cells show the change in integrity of mPTP. (d) Cardiomyocyte H_2_O_2_ concentration. (e) Cardiomyocyte superoxide anion production. (f) Cardiomyocyte LDH release. (g) Cardiomyocyte cell viability. All values are presented as the mean ± SEM, *n* = 6/group. ^▲^*p* < 0.05 versus HG group, ^∗^*p* < 0.05 versus HGHR group.

Additional investigations were performed using embryonic rat cardiomyocyte-derived H9c2 cells. TubA was administered 24 h before hypoxia induction. H/R injury noticeably induced cellular injury and oxidative stress in H9c2 cells. HDAC6 inhibition significantly decreased the apoptosis rate (Figure 6(b)), the degree of mPTP opening (Figure 6(c)), and LDH release (Figure 6(f)) but increased cell viability (Figure 6(g)) in H9c2 cells subjected to H/R under HG conditions. HDAC6 inhibition also reduced oxidative stress, as demonstrated by decreased ROS fluorescence intensity (Figure 6(a)), H_2_O_2_ concentration (Figure 6(d)), and O_2_^−^ production (Figure 6(e)).

### 3.8. Effects of TubA on HDAC6 Activity and Prdx1 Acetylation in H9c2 Cells Exposed to HG

**Figure 7:**
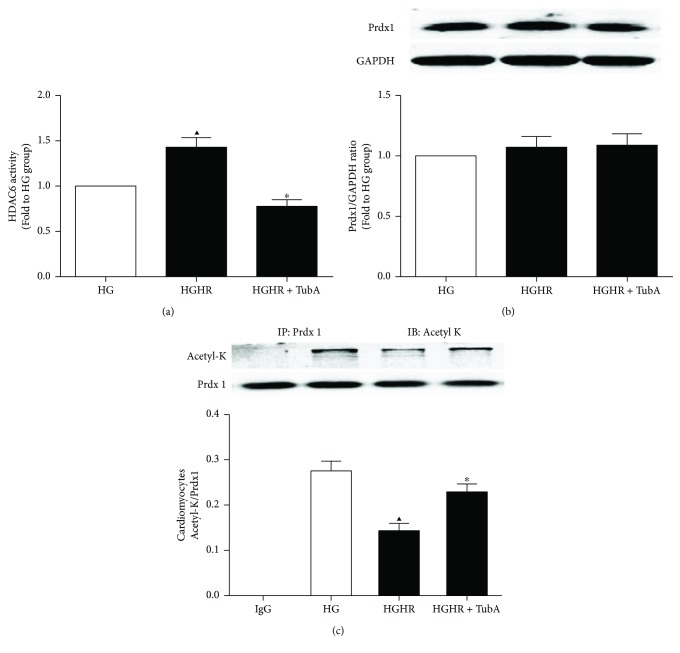
Effect of HDAC6 inhibition on HDAC6 activity, total Prdx1, and Ac-Prdx1 in H9c2 cells exposed to HG. HG: high glucose (30 mM); HR: hypoxia/reoxygenation; TubA: tubastatin A. (a) HDAC6 activity was measured by fluorometric assay kit. (b) Total Prdx1 level of H9c2 cardiomyocytes was analyzed by Western blot. GAPDH served as the loading control. (c) Acetylation level of Prdx1 was analyzed by immunoprecipitation. All values are presented as the mean ± SEM, *n* = 6/group. ^▲^*p* < 0.05 versus HG group, ^∗^*p* < 0.05 versus HGHR group.

We further determined the effect of HDAC6 activity on Prdx1 acetylation in H9c2 cells exposed to HG conditions. TubA administration significantly decreased HDAC6 activity in H9c2 cells (Figure 7(a)). H/R injury-induced HDAC6 activity (Figure 7(a)) and Prdx1 deacetylation (Figure 7(c)) in H9c2 cells were reversed by TubA administration. Additionally, H/R or HDAC6 inhibition had no effect on total Prdx1 expression (Figure 7(b)) in H9c2 cells exposed to HG conditions. These results collectively suggest that HDAC6 activity may be a modulator of Prdx1 acetylation.

### 3.9. Protective Effect of HDAC6 Inhibition Is Mediated by Prdx1 Acetylation at K197 in H9c2 Cells Exposed to HG

**Figure 8:**
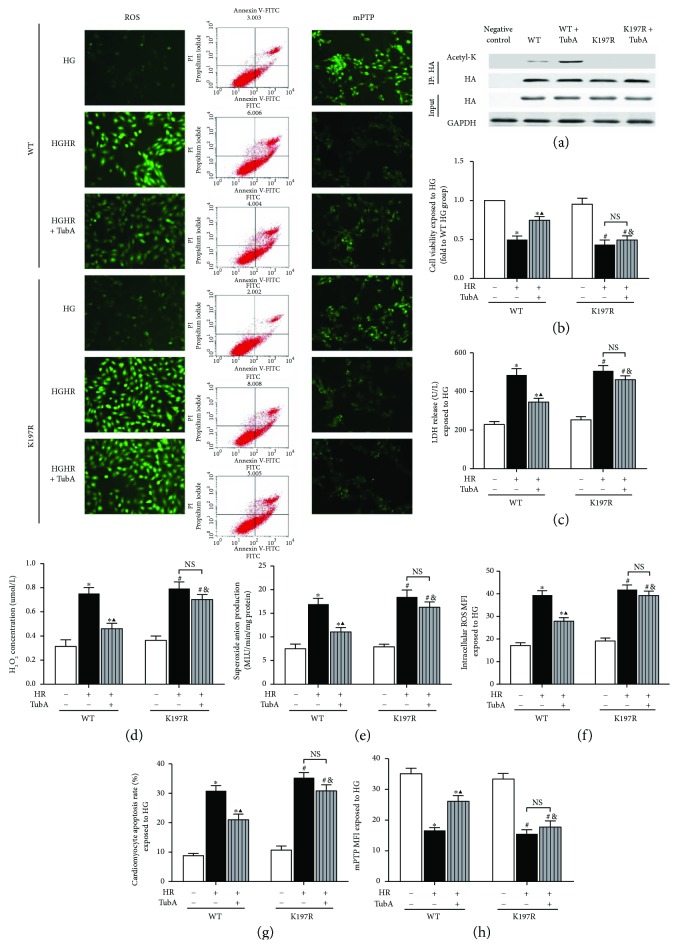
Effects of TubA on ROS, mPTP, apoptosis rate, and cellular injury in Prdx1-WT-HA- and Prdx1-K197R-HA-transfected H9c2 cells exposed to HG. HG: high glucose (30 mM); HR: hypoxia/reoxygenation; TubA: tubastatin A; WT: Prdx1-WT-HA-transfected H9c2 cells; K197R: Prdx1-K197R-HA-transfected H9c2 cells. (a) Representative immunoblot showing expression of Acetyl-K and HA in Prx1-WT- or Prdx1-K197R-transfected H9C2 cells. GAPDH served as the loading control. (b) Cardiomyocyte cell viability. (c) Cardiomyocyte LDH release. (d) Cardiomyocyte superoxide anion production. (e) Cardiomyocyte superoxide anion production. (f) Representative images of ROS staining and the mean fluorescence intensity of DCFDA in each group. (g) Representative images of flow cytometry and cardiomyocyte apoptosis rates in each group. (h) Fluorescent images of the cells show the change in integrity of mPTP. All values are presented as the mean ± SEM, *n* = 6/group. ^∗^*p* < 0.05 versus WT-HG group, ^▲^*p* < 0.05 versus WT-HGHR group, ^#^*p* < 0.05 versus K197R-HG group, and ^&^*p* < 0.05 versus WT-HGHR + TubA group.

To confirm the role of acetylated Prdx1 in the TubA-induced protective effect on H/R injury in cultured H9c2 cells exposed to HG, we constructed Prdx1 acetyl-silencing mutants with an HA tag (K197R). The expression level of the HA tag was similar in each group (Figure 8(a)). A small amount of HA-tagged Prdx1 remained constitutively associated with Acetyl-K under basal conditions, whereas TubA treatment significantly increased HA-tagged acetylated-Prdx1 levels. However, the acetyl-silenced mutant groups showed no significant association between Acetyl-K and Prdx1 (Figure 8(a)). To evaluate the role of Prdx1 in the protective effects of TubA, we transfected H9c2 cells with Prdx1-WT-HA or Prdx1-K197R-HA. We found that TubA treatment significantly decreased the apoptosis rate (Figure 8(g)), the degree of mPTP opening (Figure 8(h)), and LDH release (Figure 8(c)) but increased cell viability (Figure 8(b)) in WT-Prdx1-transfected H9c2 cells exposed to HGHR. Moreover, TubA attenuated ROS generation, as demonstrated by decreased DCFH-DA fluorescence intensity (Figure 8(f)), H_2_O_2_ concentration (Figure 8(d)), and O_2_^−^ production (Figure 8(e)). Even though TubA was administered in acetyl-silencing mutant-transfected H9c2 cells, the increase in ROS levels induced by HGHR was not rescued, unlike in the WT-Prdx1-transfected cells, which showed restored ROS levels with TubA treatment. In addition, the beneficial effect of TubA on cell viability, LDH release, mPTP opening, and cell apoptosis was abrogated in the acetyl-silencing mutant- (K197R-) transfected group. Taken together, the results of the Prdx1 mutant study indicate that the ROS-reducing activity of Prdx1 is dependent on acetylation of the K197 site. HDAC6 inhibitor modulates acetylation at K197 of Prdx1, thus contributing to cardiomyocyte survival.

## 4. Discussion

In the present study, we demonstrated that excessive ROS accumulation in diabetic hearts is accompanied by increased HDAC6 activity and decreased Prdx1 acetylation. Inhibition of HDAC6 activity by TubA *in vivo* and *in vitro* attenuates MI/R- or H/R-induced ROS generation and cellular injury. However, the protective effect of HDAC6 inhibition was partly abrogated in Prdx1 nonacetylated mimic mutant (K197R) cardiomyocytes, suggesting that HDAC6 alleviates H/R-induced O_2_^−^ and H_2_O_2_ accumulation by modulating Prdx1 acetylation at K197. Therefore, we determined a novel role for HDAC6 activity in response to oxidative stress in diabetic hearts. To our knowledge, this was the first study to examine the mechanism of cardioprotection by HDAC6 inhibition in diabetic hearts.

It is well documented that the delicate balance between the generation and scavenging of oxide free radicals due to increased ROS production and inadequate antioxidant defenses is disrupted in DM [30, 31]. Our current results show that increased cardiac O_2_^−^ and H_2_O_2_ production is concomitant with a significant increase in HDAC6 activity in STZ-induced diabetic rats. Although the precise mechanisms by which hyperglycemia induces HDAC6 activity in myocardium are not fully understood, evidence supports a vital role for HDAC6 in the stress response. For example, the catalytic activity of HDAC6 was found to be consistently increased in stressed myocardium [20]. HDAC6 inhibition leads to strong preservation of systolic function in pressure-overloaded mouse hearts [21]. Similarly, activation of HDAC6 was reported to induce a loss of contractile function by deacetylating *α*-tubulin [22]. Based on these results, we hypothesized that excessive HDAC6 activity may be responsible for aggravated MI/R injury under diabetic conditions.

To confirm whether inhibition of HDAC6 activity could alleviate MI/R injury under hyperglycemia conditions, HDAC6 activity was inhibited by TubA *in vivo* and *in vitro*. TubA is a potent, selective HDAC6 inhibitor that displays superior selectivity for the HDAC6 isozyme compared to other HDAC inhibitors and was reported to exhibit over 1000-fold selectivity for the HDAC6 isozyme compared to other highly homologous HDAC isoforms [32]. A single systemic administration of TubA *in vivo* (10 mg/kg) resulted in a 268% increase in the acetylation of *α*-tubulin, a specific substrate of HDAC6, in mouse hearts [33]. Additionally, unlike other HDAC inhibitors, the HDAC6 inhibitor does not appear to be associated with any toxicity effects, such as nausea, thrombocytopenia or fatigue, making HDAC6 an excellent therapeutic target [34]. Several studies have demonstrated the therapeutic promise of TubA in disease models, including Alzheimer's disease [35], heart remodeling [21], and kidney injury [18, 36]. In the current study, we intraperitoneally administered TubA (10 mg/kg/day) or vehicle (0.5% DMSO in 0.9% saline) daily for 1 week and observed a significant decrease in HDAC6 activity in the diabetic hearts. Our study showed that 1 week of TubA administration significantly alleviated MI/R-induced injury in diabetic hearts, as evident from a reduced infarct size, decreased serum biomarker levels, and preserved cardiac function. Moreover, TubA accelerated the elimination of O_2_^−^ and H_2_O_2_ induced by MI/R and attenuated lipid peroxidation in diabetic myocardium. Our *in vitro* study showed that inhibition of HDAC6 activity in the H9c2 cell line alleviated H/R-induced cellular injury, ROS generation, and oxidative stress. Of note, Aune et al. reported no significant recovery in the parameter of left ventricular contractile function in hearts treated with a HDAC6 inhibitor [37]. However, our study differed with that of Aune et al. in several key areas. First, the study by Aune et al. was performed in isolated nondiabetic rat hearts using a Langendorff perfusion apparatus, while our *in vivo* study was performed by occluding the LAD coronary artery in diabetic hearts. Second, diabetic cardiomyopathy is characterized by chronic structural and functional alterations [38]. As the pathogenesis of diabetic cardiomyopathy is a complicated and chronic process, acute administration of TubA has a limited effect on heart protection in diabetic rats; therefore, we chose to intraperitoneally administer TubA for one week to partially compensate for this potential problem. Furthermore, in a spinal hypoxia model, Su et al. demonstrated that knockdown of HDAC6 by siRNA accelerated ROS generation and cell apoptosis in response to hypoxia [39]. These inconsistent effects of HDAC6 inhibition may mostly be attributable to the differences in animal species, models, and methods. In addition, HDAC6 regulates various important cellular processes under physiological conditions; thus, knockdown of HDAC6 by siRNA tends to have an adverse effect on H9c2 cardiomyocytes.

Previous studies have shown that HDAC6 is a promising therapeutic target for the treatment of pressure overload in the heart, neurodegenerative disorders, brain ischemic stroke, and kidney injury, which emphasizes the role of *α*-tubulin, a well-known substrate of HDAC6, in preserving microtubule structure [40–43]. In the present study, we determined a novel role for HDAC6 in attenuating MI/R-induced oxidative stress by modulating the acetylation of Prdx1. Prdx1 degrades peroxides through reversible oxidation of their active cysteine site and plays an important role in various diseases [44–46]. Our *in vivo* study shows that excessive HDAC6 activity is concomitant with decreased Prdx1 acetylation and increased ROS generation in STZ-induced diabetic rats. Given that Prdx1 is a substrate of HDAC6, we speculate that the reduction in acetylated Prdx1 in diabetic hearts might result from elevated HDAC6 activity. As expected, inhibition of HDAC6 activity significantly increased the levels of acetylated Prdx1 and attenuated MI/R-induced ROS generation in diabetic hearts. Consist with the study by Shi et al., our result demonstrated that compared with nondiabetic hearts, there are no significant changes in Prdx1 expression in diabetic hearts [47]. Therefore, regulation of Prdx1 acetylation levels, rather than Prdx1 expression itself, is more important for regulating ROS in diabetic rats. To determine the role of HDAC6-mediated Prdx1 deacetylation in ROS generation, we constructed Prdx1 WT and acetyl-silencing mutants (K197R) by substituting arginine (R) for lysine (K) at K197. In the silencing mutant study, we showed that the protective effect of TubA was abrogated in acetyl-silencing mutant H9c2 cells. Taken together, these data suggest that increased acetylation at K197 of Prdx1 by HDAC6 inhibition contributes to decrease H/R-induced ROS generation in cardiac H9c2 cells exposed to high glucose.

Clinical management of type 1 DM mainly focuses on blood glucose control. However, approaches to prevent and treat cardiovascular complications have been largely extrapolated from studies on type 2 diabetes [48]. However, type 2 diabetes accounts for more than 90% of diabetes cases [49]. In the present study, the STZ-induced diabetic rat model more closely parallels type 1 diabetes. Further studies of HDAC6 in type 2 diabetic rats are necessary. Uncontrolled chronic hyperglycemia leads to severe diabetic cardiomyopathy. Our experimental findings are only instructive for myocardial protection in severe hyperglycemic conditions. As insulin is the first treatment of choice for diabetic patients with uncontrolled hyperglycemia, further studies are needed to determine whether insulin acts synergistically with TubA or counteracts the advantages elicited by TubA in the diabetic heart. Moreover, the present study showed that HDAC6 inhibition-mediated cardioprotection in diabetic hearts was related to redox regulation. Previous studies have shown that HDAC6 is involved in ubiquitination and autophagy through binding Parkin, *α*-tubulin, or P62 [50–52]. Further studies are necessary to better define whether HDAC6 inhibition attenuates MI/R injury by improving autophagy or modulating protein ubiquitination.

Taken together, our study demonstrates that excessive HDAC6 activity under diabetic conditions contributes to aggravated MI/R injury in diabetic hearts. Pharmacological inhibition of HDAC6 activity *in vivo* and *in vitro* restored the ROS elimination capacity of Prdx1 by modulating acetylation at K197. As there are no statistically significant changes in Prdx1 levels in diabetic hearts, acetylation of Prdx1 is vital for its peroxide-reducing capacity. Our findings suggest that HDAC6-mediated Prdx1 acetylation might be a promising therapeutic strategy for MI/R in diabetic hearts. However, the precise mechanism requires further study in HDAC6 knockout or knock-in mice.

## Figures and Tables

**Table 1:** General characteristics of each group after 8 weeks.

Parameters/Group	N	D	D + TubA
Blood glucose (mM)	6.27 ± 0.78	28.52 ± 2.95^∗^	26.13 ± 3.18^∗^
Body weight (g)	479.5 ± 15.31	254.8 ± 19.45^∗^	265.4 ± 21.7^∗^
Water intake (ml/kg/day)	107.0 ± 7.9	855.0 ± 93.4^∗^	826.0 ± 85.4^∗^
Food consumption (g/kg/day)	68. 3 ± 5.6	193.9 ± 20.1^∗^	187.3 ± 18.5^∗^

N: nondiabetic rats; D: STZ-induced diabetic rats; and D + TubA: tubastatin A (10 mg/kg) administered daily by i.p. injection for 1 week in diabetic rats. The results are expressed as the mean ± SEM. ^∗^*p* < 0.05 versus the N group; *n* = 12.
